# Supplementary material for: BERMUDA: a novel deep transfer learning method for single-cell RNA sequencing batch correction reveals hidden high-resolution cellular subtypes
Source: Genome Biol. 2019 Aug 12;20:165. doi: 10.1186/s13059-019-1764-6 (PMC6691531; doi:10.1186/s13059-019-1764-6)
Supplement: Supplementary file 1 — Supplementary figures and tables (PDF 3839 kb) [file 13059_2019_1764_MOESM1_ESM.pdf]

Supplementary information of

BERMUDA: a novel deep transfer learning method for single-cell RNA sequencing batch correction reveals hidden high-resolution cellular subtypes

Supplementary figures

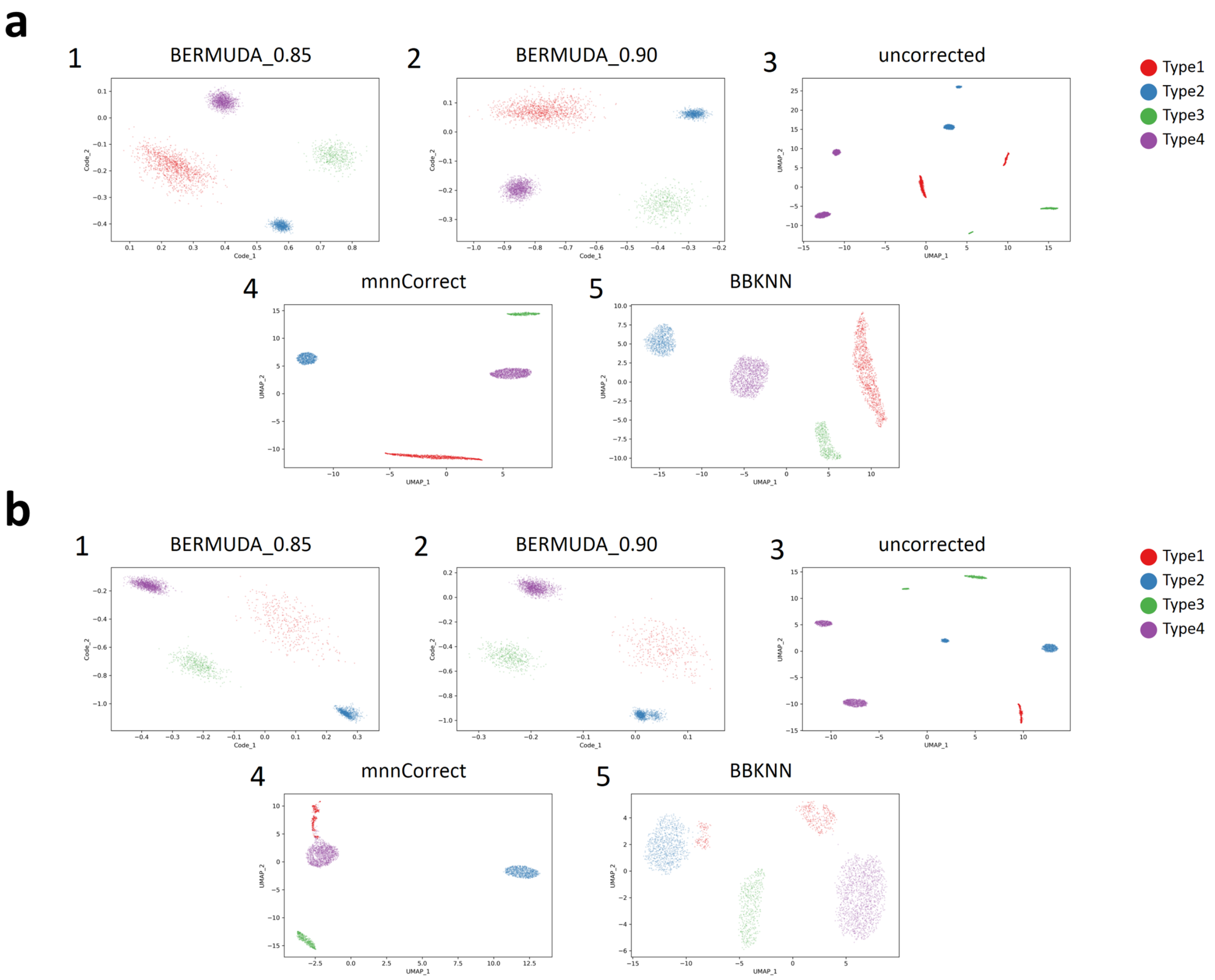

Figure S1. Removing batch effects in simulated data generated by 2-dimensional Gaussian distribution. Visualizations of results for simulated data generated by 2-dimensional Gaussian distribution. Results of our method are visualized by the 2-dimensional code in the trained autoencoder, while results of other methods are visualized using UMAP. BERMUDA _0.85 and BERMUDA_0.90 represent our method with $S_{thr} = 0.85$ and 0.90 respectively. a. Results of *Experiment all*. b. Results of *Experiment removal1*.

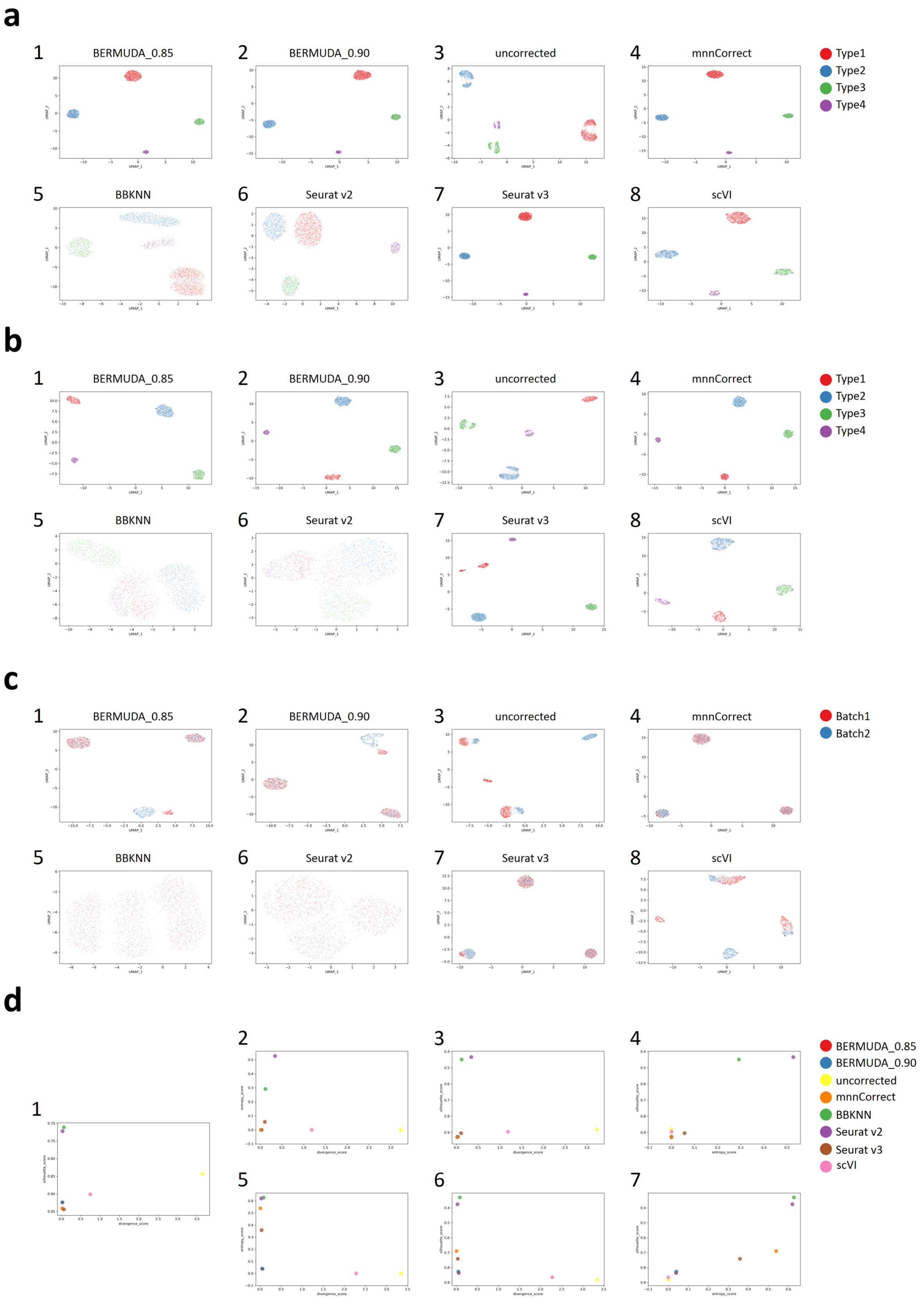
a
1 BERMUDA_0.85
2 BERMUDA_0.90
3 uncorrected
4 mnnCorrect
5 BBKNN
6 Seurat v2
7 Seurat v3
8 scVI
Type1
Type2
Type3
Type4
b
1 BERMUDA_0.85
2 BERMUDA_0.90
3 uncorrected
4 mnnCorrect
5 BBKNN
6 Seurat v2
7 Seurat v3
8 scVI
Type1
Type2
Type3
Type4
c
1 BERMUDA_0.85
2 BERMUDA_0.90
3 uncorrected
4 mnnCorrect
5 BBKNN
6 Seurat v2
7 Seurat v3
8 scVI
Batch1
Batch2
d
1
2
3
4
5
6
7
BERMUDA_0.85
BERMUDA_0.90
uncorrected
mnnCorrect
BBKNN
Seurat v2
Seurat v3
scVI

Figure S2. Removing batch effects in simulated data generated by Splatter. a. UMAP visualizations of results for *Experiment all* colored by cell types. b. UMAP visualizations of results for *Experiment removal1* colored by cell types. c. UMAP visualizations of results for *Experiment removal2* colored by batches. d. Evaluation of batch correction performance on Splatter dataset using the proposed metrics. The $silhouette\_score$ axis is reversed so that points close to the bottom-left corner indicate better results. c1. *Experiment all*. c2-4. *Experiment removal1*. c5-7. *Experiment removal2*.

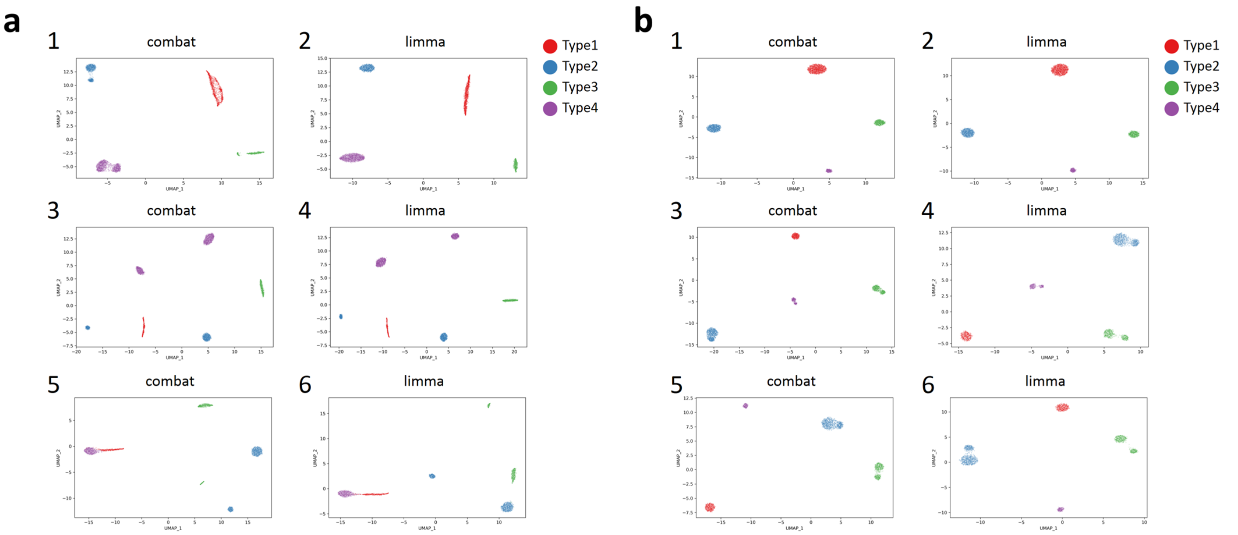

Figure S3. *Combat* and *limma* fail to correctly remove batch effects in simulated datasets. *Combat* and *limma* could not remove batch effects in scRNA-seq data correctly when not all cellular states were shared by all the batches (*Experiment removal1* and *Experiment removal2*). a. UMAP visualizations of batch correction results for simulated data generated by 2-dimensional Gaussian distribution. a1-2. *Experiment all*. a3-4. *Experiment removal1*. a5-6. *Experiment removal2*. b. UMAP visualizations of batch correction results for simulated data generated by Splatter. b1-2. *Experiment all*. b3-4. *Experiment removal1*. b5-6. *Experiment removal2*.

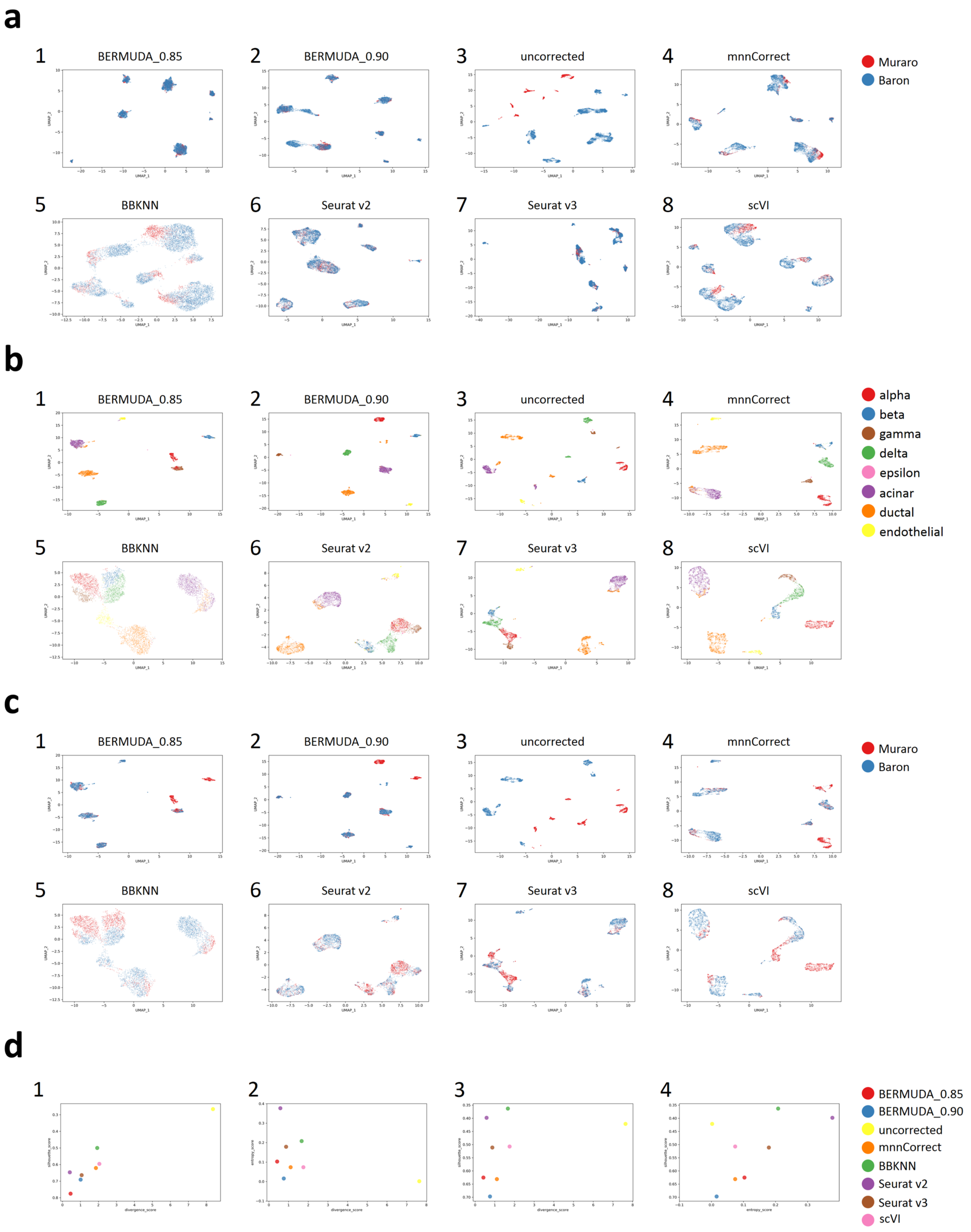

Figure S4. Removing batch effects in scRNA-seq data of pancreas cells with *Muraro batch* and *Baron batch*. a. UMAP visualizations of results for *Experiment all* colored by batches. b. UMAP visualizations of results for *Experiment removal* colored by cell types. c. UMAP visualizations of results in

Experiment removal colored by batches. d. Evaluation of batch correction performance on *Experiment removal* using the proposed metrics. The $silhouette\_score$ axis is reversed so that points close to the bottom-left corner indicate better results. d1. *Experiment all*. d2-4. *Experiment removal*.

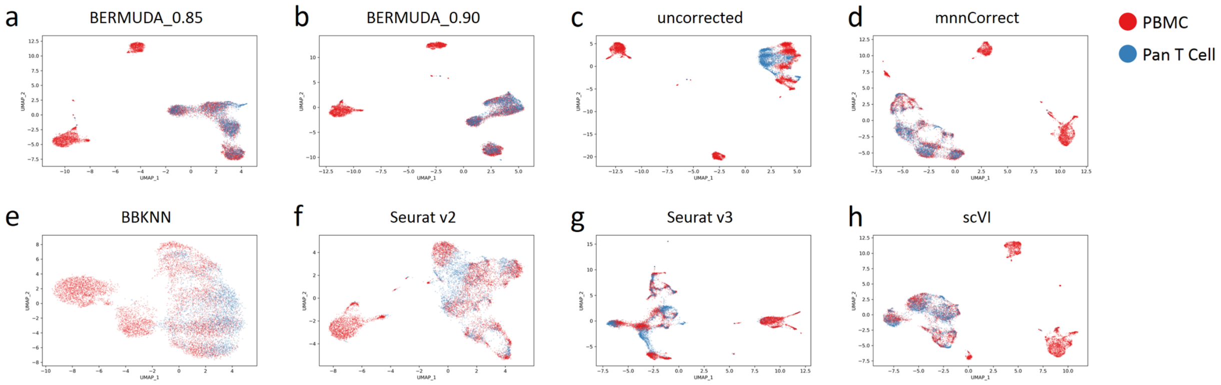

Figure S5. Removing batch effects in scRNA-seq data of PBMCs. UMAP visualizations of results on PBMC dataset colored by batches. Our method correctly merged T cells from the both batches, while preserved the structures of cell clusters specific to the *PBMC batch*.

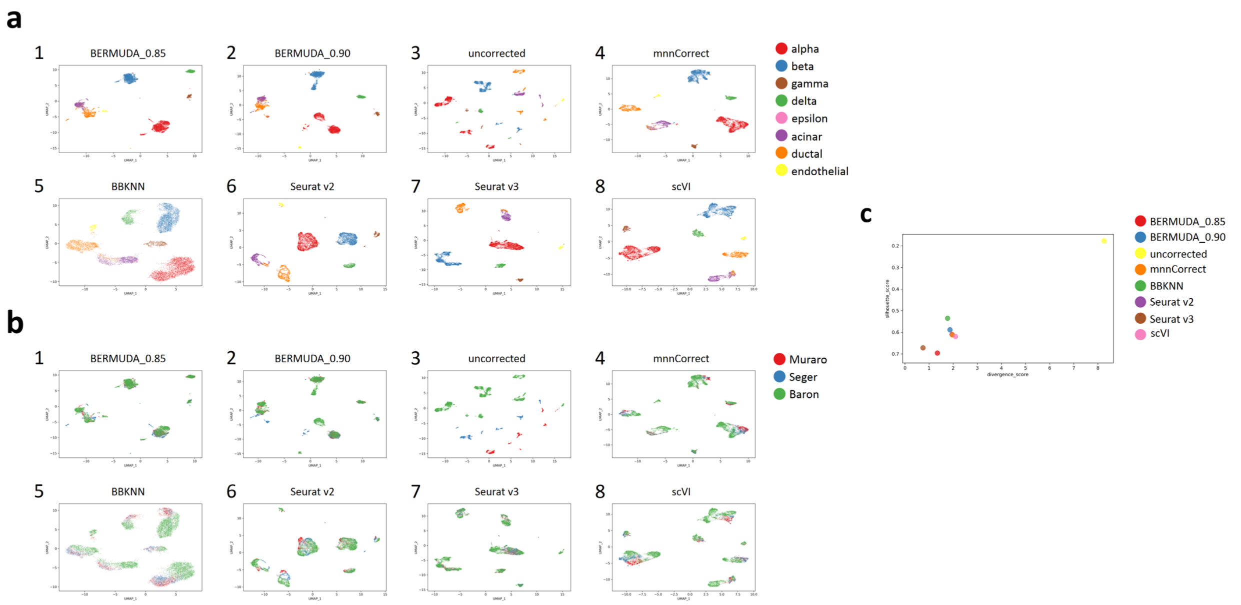

Figure S6. Removing batch effects in scRNA-seq data of pancreas cells with multiple batches. All the cells from *Muraro batch*, *Baron batch*, and *Segerstolpe batch* were used for analysis. a. UMAP visualizations of results for *Experiment all* colored by cell types. b. UMAP visualizations of results for *Experiment all* colored by batches. c. Evaluation of batch correction performance on *Experiment all* using the proposed metrics.

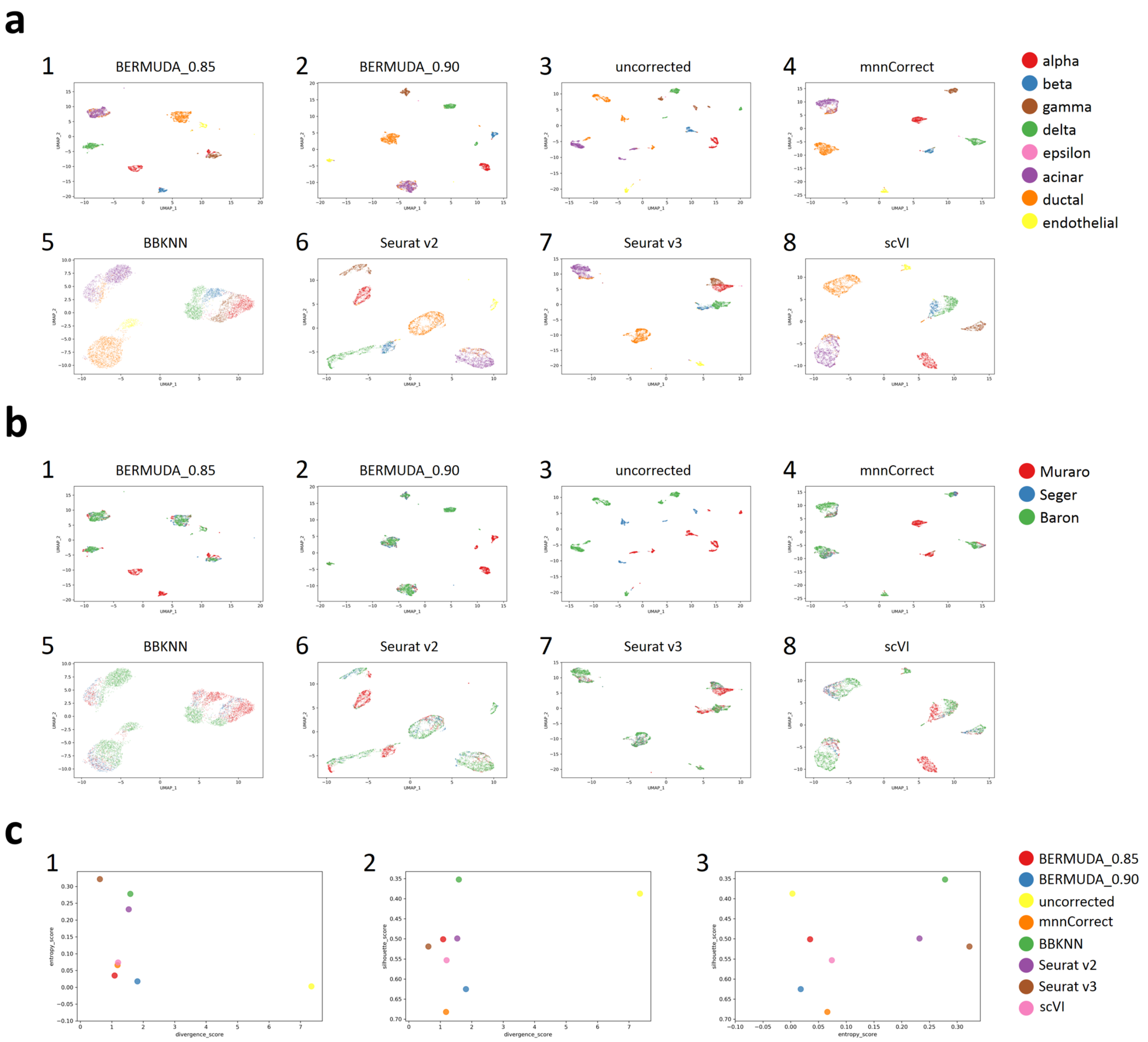

Figure S7. Removing batch effects in scRNA-seq data of pancreas cells with multiple batches of different cell population compositions. Alpha and beta cells from *Baron batch* and *Segerstolpe batch* were removed from analysis. a. UMAP visualizations of results for *Experiment removal* colored by cell types. b. UMAP visualization of results for *Experiment removal* colored by batches. c. Evaluation of batch correction performance on *Experiment removal* using the proposed metrics.

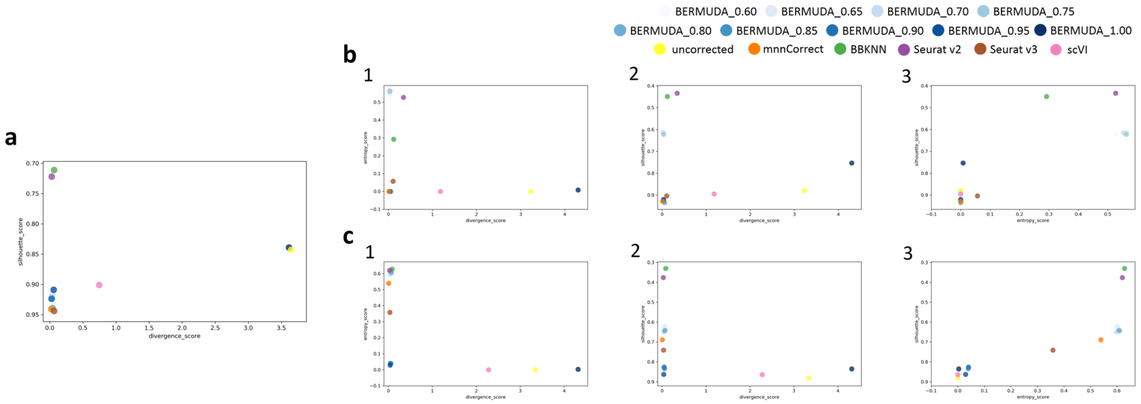

Figure S8. Results of Splatter dataset using different S_{thr} values evaluated by proposed metrics. a. *Experiment all*. b. *Experiment removal1*. c. *Experiment removal2*. The most important parameter in our method is S_{thr}, which is the threshold applied on the cluster similarity score to identify similar clusters across different batches. S_{thr} can affect the results of batch correction, where a lower S_{thr} value can produce a more homogeneous mixture of different batches within cell types and a higher S_{thr} value can help to retain more batch-specific biological signals. We experimentally demonstrate our choice of S_{thr} by evaluating the performance of our method using S_{thr} ranges from 0.60 to 1.00. Our method consistently outperformed the existing methods on the Splatter dataset when choosing S_{thr} between 0.85 and 0.90. More specifically, when all the cell types were shared in different batches, generally $S_{thr} \leq 0.9$ produced competitive results, since we did not need to consider the case where different cell types might be mixed together by using a low threshold. However, when we introduced large differences in cell population compositions by removing cell types from specific batches, we observed that S_{thr} between 0.85 and 0.90 consistently produced best results across different experiments, and we used S_{thr} between 0.85 and 0.90 as a default parameter choice for *BERMUDA*.

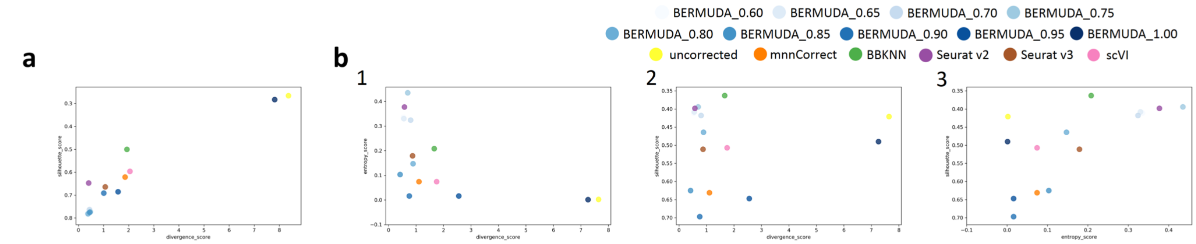

Figure S9. Results of pancreas dataset with *Muraro batch* and *Baron batch* using different S_{thr} values evaluated by proposed metrics. a. *Experiment all*. b. *Experiment removal*. Our method consistently outperformed existing methods on the pancreas dataset when choosing S_{thr} between 0.85 and 0.90.

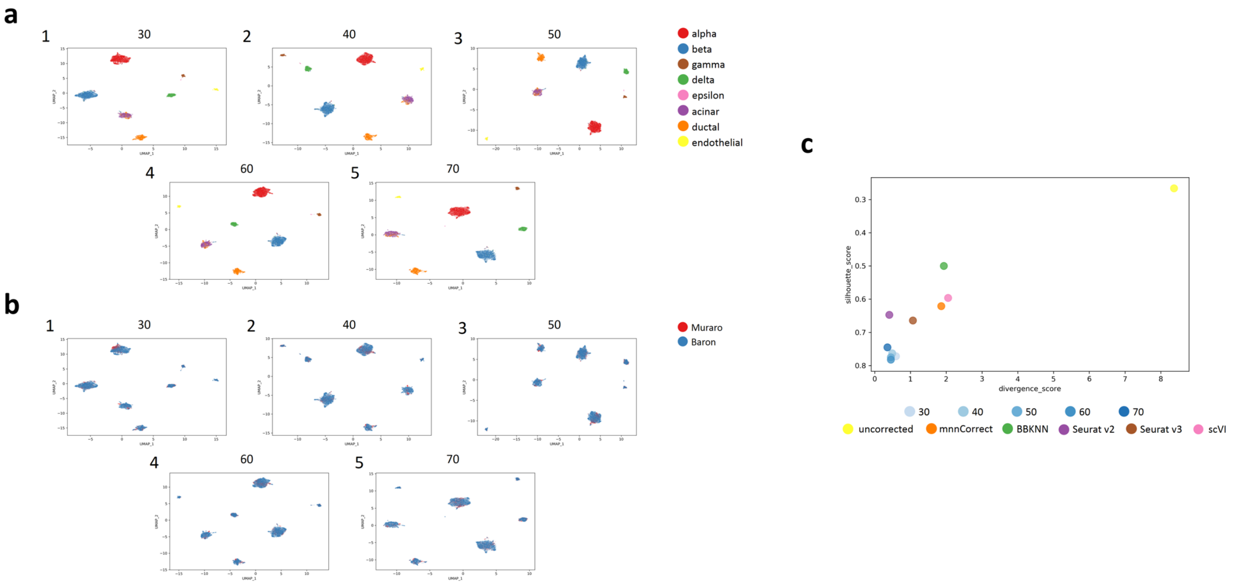

Figure S10. *BERMUDA* is robust to the number of cells sampled from each cluster in a mini-batch. The number of cells sampled from each cluster in a mini-batch, n_{mb}, is a hyperparameter in *BERMUDA*. We experimentally demonstrate that *BERMUDA* is robust to the choice of n_{mb} by evaluating the performance of *BERMUDA* using n_{mb} ranges from 30 to 70. The results were generated by performing *Experiment all* to combine *Muraro batch* and *Baron batch* in the pancreas dataset with $S_{thr} = 0.85$. We observed that the performance of *BERMUDA* was insensitive to the choice of n_{mb} and *BERMUDA* consistently outperformed existing methods under a wide range of n_{mb} values. a. UMAP visualizations of results colored by cell types. The number above each figure represents the value of n_{mb} used for training *BERMUDA*. b. UMAP visualizations of results colored by batches. c. Evaluation of batch correction performance using the proposed metrics.

Supplementary tables

Table S1. List of differently expressed genes within alpha cells in the pancreas dataset.

Gene Symbol	Adjusted p-value (Muraro vs. Baron)	Adjusted p-value (Baron vs. Baron)
PCSK1N	0.00E+00	7.97E-55
FAP	0.00E+00	6.04E-76
G6PC2	7.31E-290	3.54E-168
SLC30A8	5.80E-281	2.75E-162
SLC38A4	1.53E-277	7.67E-51
ARRDC4	5.64E-249	1.88E-60
ABCC8	6.70E-241	2.05E-130
TM4SF4	1.06E-173	8.14E-221
CRYBA2	1.00E-169	1.86E-202
**MAFB**	**1.42E-158**	**4.16E-109**
**ARX**	**1.09E-145**	**3.21E-60**
TXNIP	4.58E-104	6.57E-122
INSIG1	9.34E-95	9.48E-53
FXYD6	1.41E-93	6.77E-119
XIST	6.48E-85	1.19E-169
SERPINA1	1.62E-76	3.09E-52
S100A11	7.41E-74	7.02E-87
TNFRSF12A	1.38E-69	6.32E-94
ANXA2	1.06E-64	1.45E-111

Only genes with adjusted p-value $\leq 10^{-50}$ in both tests are reported. The bold genes are discussed in detail in the paper.

Table S2. List of differently expressed genes within beta cells in the pancreas dataset.

Gene Symbol	Adjusted p-value (Muraro vs. Baron)	Adjusted p-value (Baron vs. Baron)
SYT13	6.98E-194	2.14E-84
SLC30A8	1.34E-179	2.47E-223
ABCC8	2.05E-170	1.95E-162
G6PC2	3.16E-162	5.89E-155
INS	3.93E-160	5.62E-83
**PDX1**	**1.35E-151**	**8.21E-51**
**MAFB**	**1.06E-150**	**6.35E-95**
**MAFA**	**9.22E-135**	**2.77E-54**
PLCXD3	7.72E-126	1.97E-62
PCSK1	1.90E-97	7.74E-96
WNT4	1.11E-81	1.27E-61
TIMP2	4.85E-80	1.90E-61
EDN3	5.89E-62	4.68E-92

Only genes with adjusted p-value $\leq 10^{-50}$ in both tests are reported. The bold genes are discussed in detail in the paper.

Table S3. Running time of different methods in the pancreas dataset.

Method	Running time(s)
BERMUDA ($S_{thr} = 0.85$)	285.91
BERMUDA ($S_{thr} = 0.90$)	262.98
mnnCorrect	35.90
BBKNN	5.02
Seurat v2	338.33
Seurat v3	75.27
scVI	360.33

The running time was measured by performing *Experiment all* on *Muraro batch* and *Baron batch*. Since we expect *BERMUDA* to be adopted by biologists who may not always have easy access to high-end computing facilities, and some of the methods compared do not have a GPU implementation, we evaluated the running time on a desktop computer with a CPU (2.7 GHz Intel Core i5) for fair comparison.
