# Supplementary material for: BERMUDA: a novel deep transfer learning method for single-cell RNA sequencing batch correction reveals hidden high-resolution cellular subtypes
Source: Genome Biol. 2019 Aug 12;20:165. doi: 10.1186/s13059-019-1764-6 (PMC6691531; doi:10.1186/s13059-019-1764-6)
Supplement: Supplementary file 2 — Review history (DOCX 44 kb) [file 13059_2019_1764_MOESM2_ESM.docx]

Review History

**First round of review**

**Reviewer 1**

**Were you able to assess all statistics in the manuscript, including the appropriateness of statistical tests used?**

Yes: The statistical tests are appropriate.

**Were you able to directly test the methods?**

No

**Comments to author:**

Single cell RNA-seq has been widely adopted in broad applications. Removing batch effect is critical for comparing and analyzing data generated in different batches. In this well-written manuscript, the authors proposed a new method using autoencoder for this purpose. Compared with the other existing methods, this approach utilizing the cluster similarity rather than common nearest neighbor to align different batches. The authors have conducted thorough assessment of the method and comparison with the existing methods. They showed BERMUDA outperformed the other popular methods of Seurat v2/v3, mnnCorrect, BBKNN. Importantly, BERMUDA can preserve the batch-specific biological signals, which is crucial in combining data from different batches for an integrative analysis. I have some minor comments for the authors to consider for improving the clarity of the manuscript.

1. In Results, the description of BERMUDA, a brief description with more details of how the recontruction loss is combined with the transfer loss in training audoencoder would make it easier for readers to understand the method. The description in Methods is unclear about using mini-batch of calculating the loss functions. How to decide the mini-batches? Are the 50 cells randomly selected from each cluster? Or the cells closest to the center of the clusters are selected? Can they comment on the advantage of using MMD instead of other similarity metrics?

2. Considering the similarity between shared clusters in different batches is a great idea. Clustering is thus a critical step for the success of BERMUDA. Or is the result insensitive to how the data are clustered in the two batches? For some data, the clusters may not be well separated due to technical or biological noise. It'd be helpful for users to get some guidance of how to handle such data.

3. The UMAP clustering results are intuitive to illustrate the results from different methods. It would be informative to show the divergence and entropy scores in supplementary.

4. Can the authors show the running time for each method?

Minor typos:

Page 17 line 427, the reference is missing.

**Reviewer 2**

**Were you able to assess all statistics in the manuscript, including the appropriateness of statistical tests used?**

**Yes,** I think the proposed neural net model is intuitive and reasonable.

**Were you able to directly test the methods?**

No.

**Comments to author:**

The paper proposed an approach for correcting batch effects from scRNA-seq data using deep (variational) autoencoder. The main contribution is the introduction of MMD (Maximum Mean Discrepancy; A. Gretton et al.) in the objective function to optimize the difference between latent features (low dimensional representation) of batches. The approach of this method is intuitive, but the overall presentation of the analysis is not clear enough to properly assess the performance.

1.	Figure quality in the main pdf file is seriously low. I cannot read what is on x-axis and y-axis. It is difficult to properly assess the performance. (The authors may have deliberately enhanced figure resolution (300dpi) as per journal's guideline, but in my opinion, this rule should be applied to only camera images, not the computer-based plots.)

2.	scVI (Nat. Methods, 2018) is also able to correct batch effects under deep learning framework. It would be great if the authors include scVI in their comparison and discuss pros and cons.

3.	I found many typos and grammatical errors. The manuscript needs English editing. BURMUDA  BERMUDA in both main text and supplement. Line 142, 4-5 5-6

4.	Line 183-184, 407-408 need to be rephrased.

5.	Data analysis methods for both simulation and real data are very briefly described, and it is difficult to understand what is going on. The overall analysis methods including scenarios should be described in detail. For example, line 107, 108, Does 'holding out' mean leaving or removing? This is very confusing expression; from line 165, it seems to mean removing.

6.	Line 172-173, alpha and beta are from Muraro batch?

7.	While mnnCorrect selects mutually nearest neighbors between batches, the proposed method chooses the nearest neighbor based on only one direction (line 366 - 369). I wonder what the effect of this loosened criterion is? If a cluster is specific to only one batch, it still finds its counterpart cluster in the other batch, and this similarity value is reflected in L2(X,X') function potentially causing some undesirable effect. I wonder why the authors did not use only mutually nearest neighbors.

8.	For autoencoder training, 50 cells were used for each cell type. Justification of this choice is necessary; for example, effect of using the same number of cells as compared to using disproportionate number of cells in training. How about using max(50, min.number of cells in each type)?

9.	Line 403, how the lambda was increased during training?

10.	An error in line 427.

**Authors response to reviewers**

# Response to Reviewer 1

**Comment 1**: *In Results, the description of BERMUDA, a brief description with more details of how the recontruction loss is combined with the transfer loss in training audoencoder would make it easier for readers to understand the method. The description in Methods is unclear about using mini-batch of calculating the loss functions. How to decide the mini-batches? Are the 50 cells randomly selected from each cluster? Or the cells closest to the center of the clusters are selected? Can they comment on the advantage of using MMD instead of other similarity metrics?*

**Response:** We thank the reviewer for this comment. We have rewritten the Results section to include more information about the loss function used in training *BERMUDA* (Line 87-96).

We used the *mini-batch gradient descent* algorithm in training *BERMUDA*, which is widely adopted in training neural networks. For each iteration within each epoch during the training process, we sampled 50 cells from each identified cell cluster to construct a “mini-batch.” The 50 cells were randomly sampled from each cluster. Each epoch included multiple iterations to cover all the cells in the entire dataset. We have updated the Methods section accordingly to include detailed information of the training procedure (Line 439-447).

MMD is a non-parametric distance estimate between distributions based on the reproducing kernel Hilbert space (RKHS). MMD has proven to be highly effective in many deep transfer learning tasks [1-4]. Since MMD does not require density estimates as an intermediate step and does not assume any parametric density on the data, it can be applied to different domains [5]. MMD is also memory- efficient, fast to compute, and performs well on high dimensional data with low sample size [6, 7]. We have also updated the Results section to include the advantages of using MMD-based transfer loss (Line 454-458).

**Comment 2**: *Considering the similarity between shared clusters in different batches is a great idea. Clustering is thus a critical step for the success of BERMUDA. Or is the result insensitive to how the data are clustered in the two batches? For some data, the clusters may not be well separated due to technical or biological noise. It'd be helpful for users to get some guidance of how to handle such data.*

**Response:** Thank you for this insightful comment. While *BERMUDA* was originally designed with a focus on scRNA-seq data with distinct cell populations, it can also accommodate data in which the clusters are not well separated in the uncorrected dataset. Specifically, under the current framework of *BERMUDA*, such data can be handled by increasing the resolution in the graph- based clustering algorithm to align clusters at a more granular level. For example, when aligning two peripheral blood mononuclear cell batches (Figure 4), pan T cells were visualized as a	large

cluster without clear clustering structure in the uncorrected data (Figure 4c, Additional file 1: Figure S5c), but *BERMUDA* can still distinguish different cell types within pan T cells (Figure 4b). It is also the focus of our future work to improve *BERMUDA* to accommodate such data even more effectively. We have updated the Discussion section to respond to this comment (Line 344-351).

**Comment 3**: *The UMAP clustering results are intuitive to illustrate the results from different methods. It would be informative to show the divergence and entropy scores in supplementary.*

**Response:** We thank the reviewer for this great suggestion and have rearranged our figures accordingly.

**Comment 4**: *Can the authors show the running time for each method?*

**Response:** Based on the reviewer’s suggestion, we used *Experiment all* in aligning *Muraro batch* and *Baron batch* as an example to show the running time for each method (Additional file 1: Table S3). Since we expect *BERMUDA* to be adopted by biologists who may not always have easy access to high-end computing facilities, and some of the methods compared do not have a GPU implementation, we evaluated the running time on a desktop computer with a CPU (2.7 GHz Intel Core i5) for fair comparison. For reviewers’ convenience, we also list it below.

**Table S3.** Running time of different methods in the pancreas dataset.

| **Method** | **Running time(s)** |
| --- | --- |
| BERMUDA (𝑆"#$ = 0.85) | 285.91 |
| BERMUDA (𝑆"#$ = 0.90) | 262.98 |
| mnnCorrect | 35.90 |
| BBKNN | 5.02 |
| Seurat v2 | 338.33 |
| Seurat v3 | 75.27 |
| scVI | 360.33 |

**Comment 5**: *Page 17 line 427, the reference is missing.*

**Response:** We thank the reviewer pointing out this error. We have corrected it accordingly (Line 393).

# Response to Reviewer 2

**Comment 1**: *Figure quality in the main pdf file is seriously low. I cannot read what is on x-axis and y-axis. It is difficult to properly assess the performance. (The authors may have deliberately enhanced figure resolution (300dpi) as per journal's guideline, but in my opinion, this rule should be applied to only camera images, not the computer-based plots.)*

**Response:** Thank you for this suggestion. We have increased the resolution of our figures. Currently, the figures in the main pdf were generated automatically by the journal submission system, which provides access to the original high-resolution figures via the link on the upper right side of each page (*e.g.* “Click here to download Figure Figure1.png”).

**Comment 2**: *scVI (Nat. Methods, 2018) is also able to correct batch effects under deep learning framework. It would be great if the authors include scVI in their comparison and discuss pros and cons.*

**Response:** We thank the reviewer for this great suggestion. We have included the results of *scVI* in all our experiments in the revised manuscript. In the tests, *scVI* showed promising results in separating different cell types, but did not perform well in aligning cells from different batches within each cell type, resulting in high values of 𝑑𝑖𝑣𝑒𝑟𝑔𝑒𝑛𝑐𝑒_𝑠𝑐𝑜𝑟𝑒. We have updated the Results section to include the comparison with *scVI* (Line 59-61, 160-161, 174-178, 196-198, 281-282, 522-525).

**Comment 3**: *I found many typos and grammatical errors. The manuscript needs English editing. BURMUDA D BERMUDA in both main text and supplement. Line 142, 4-5 D 5-6*

**Response:** Thank you for this suggestion. We have carefully edited and revised the manuscript.

**Comment 4**: *Line 183-184, 407-408 need to be rephrased.*

**Response:** Thanks for this great suggestion. We have rewritten these parts of the manuscript (Line 214-218, 373-374).

**Comment 5**: *Data analysis methods for both simulation and real data are very briefly described, and it is difficult to understand what is going on. The overall analysis methods including scenarios should be described in detail. For example, line 107, 108, Does 'holding out' mean leaving or removing? This is very confusing expression; from line 165, it seems to mean removing.*

**Response:** We thank the reviewer for this important comment. We have provided more details regarding the analysis in the revision. Specifically, we have added a subsection titled “Compare the performance of *BERMUDA* versus existing methods under different cell population compositions” to introduce all the data analysis experiments performed in this paper (Line 107-123). We have also added a table (Table 2) to summarize the details of each data analysis experiment.

“Holding out” means removing the selected data points, which we have clarified in our manuscript accordingly. We also changed the names of the experiments (*e.g. Experiment hold* to *Experiment removal*) for less confusion. To assess the performance of *BERMUDA* under different cell population compositions across different batches, we performed multiple data analysis experiments in each simulated dataset and human pancreas dataset. In some experiments, we removed some cell types from specific batches to create different cell type compositions across different batches (*e.g. Experiment removal*).

**Comment 6**: *Line 172-173, alpha and beta are from Muraro batch?*

**Response:** Sorry for the confusion. In *Experiment removal* in this section, we removed alpha and beta cells from *Baron batch* to evaluate whether *BERMUDA* can properly align batches with vastly different cell population compositions in real data. We have rewritten this part of the manuscript to make it clearer (Line 202).

**Comment 7**: *While mnnCorrect selects mutually nearest neighbors between batches, the proposed method chooses the nearest neighbor based on only one direction (line 366 - 369). I wonder what the effect of this loosened criterion is? If a cluster is specific to only one batch, it still finds its counterpart cluster in the other batch, and this similarity value is reflected in L2(X,X') function potentially causing some undesirable effect. I wonder why the authors did not use only mutually nearest neighbors.*

**Response:** We thank the reviewer for this comment and have rewritten this section to make it clearer. To begin with, *BERMUDA* depends on the similarity between cell clusters to align different batches, rather than the similarity between pairs of cells (*e.g.* mutually nearest neighbors) as in mnnCorrect. Our loosened criterion is to accommodate the case where a cell cluster in one batch corresponds to multiple clusters in another batch, which makes *BERMUDA* more robust to the results in the clustering step.

We denote 𝑀1_2_,4_2_,1_5_,4_5_ as the similarity score between cluster 𝑗7	in batch 𝑖7 and cluster 𝑗8	in batch

𝑖8, and ::;(𝐙1_2_,4_2_, 𝐙1_5_,4_5_) as the MMD distance between the learned embeddings between cluster

𝑗7	in batch 𝑖7	and cluster 𝑗8	in batch 8 . Considering the case where cluster 1 in batch 1 is

identified to be similar to cluster 2 and 3 in batch 2 with high confidence by MetaNeighbor (*e.g.*

𝑀7,7,8,8 = 𝑀8,8,7,7 = 0.99, 𝑀7,7,8,C = 𝑀8,C,7,7 = 0.98), where cluster 2 and 3 might be separated from a single, larger cluster by the clustering algorithm. If we only considered mutual nearest clusters, we would have only aligned cluster 1 in batch 1 with cluster 2 in batch 2. However, by using our loosened criterion, according to Equation 3, we have 𝑀7,7,8,8 = 𝑀8,8,7,7 = 0.99, 𝑀7,7,8,C = 0 and

𝑀8,C,7,7 = 0.98 at first. Next, by using Equation 4 to make the similarity scores between two clusters

symmetrical, we eventually get 𝑀7,7,8,C = max (𝑀7,7,8,C, 𝑀8,C,7,7) = 0.98, which faithfully captures the similarity relationships identified.

However, if a cluster is specific to only one batch, by taking advantage of MetaNeighbor to determine the similarity between clusters, we can prevent finding a spurious counterpart for such cluster. For example, if cluster 1 from batch 1 is specific to batch 1, then 𝑀7,7,1,4 will be smaller than

𝑆"#$	for all	and , which means 𝑀7,7,1,4 = 0, ∀𝑖, 𝑗 when training *BERMUDA* (Equation 5). In this

way, cluster 1 in batch 1 will not be aligned to other clusters during the training process.

**Comment 8**: *For autoencoder training, 50 cells were used for each cell type. Justification of this choice is necessary; for example, effect of using the same number of cells as compared to using disproportionate number of cells in training. How about using max(50, min.number of cells in each type)?*

**Response:** Thank you for this insightful comment. We adopted the *mini-batch gradient descent* algorithm in training *BERMUDA*. Mini-batch gradient descent is conventional for training neural networks and multiple iterations were performed in each epoch to cover all the cells in the entire dataset when training *BERMUDA*. In each iteration of each epoch during the training process, 50 cells were sampled from each identified cell cluster to construct a “mini-batch”, which was for the gradient descent learning process. The loss calculated from this “mini-batch” was then used for back propagation to optimize the parameters for the neural network in *BERMUDA* using gradient descent. We have also made the clarification in our manuscript with detailed explanation of the training procedure (Line 439-447).

We sampled the same number of cells from each cluster to reduce the number of hyperparameters in training *BERMUDA*, making it more user-friendly. We also showed experimentally that *BERMUDA* is insensitive to the number of cells sampled from each cluster (𝑛JK) in mini-batch gradient descent and can outperform existing methods under a wide range of 𝑛JK values (Additional file 1: Figure S10).

Using *max(50, minimum number of cells in each type)* may encounter difficulties when dealing with datasets with a similar number of cells in each cluster. For example, if we have a dataset with two

batches, each containing four clusters with 1,000 cells, we will have *max(50, minimum number of cells in each type)* = 1,000 during training. The model may not be properly trained when the size of mini-batches is large in mini-batch gradient descent since it could reduce the stochasticity during the optimization process, making the model more susceptible to local optimal solutions.

**Comment 9**: *Line 403, how the lambda was increased during training?*

**Response:** Thank you for this great comment. We followed the strategy introduced by Ganin et al.

[8] to gradually increase 𝜆 from 0 to 1 during training. The regularization parameter at epoch	𝑝

can be calculated as

𝜆N

8

Q2RS

=

− 1,

7OP	TS

where 𝑛𝑝 is the number of total epochs in training. We have also clarified this in our manuscript with the details of this strategy (Line 467-473).

**Comment 10**: *An error in line 427.*

**Response:** We thank the reviewer pointing out this error. We have corrected it accordingly (Line 393).

# References

1. Ghifary M, Kleijn WB, Zhang M: **Domain Adaptive Neural Networks for Object Recognition.** In *PRICAI*. 2014: 898-904.
2. Long M, Cao Y, Wang J, Jordan MI: **Learning transferable features with deep adaptation networks.** In *ICML*. 2015:97-105.
3. Long M, Zhu H, Wang J, Jordan MI: **Deep transfer learning with joint adaptation networks.** In *ICML*. 2017:2208-2217.
4. WEI Y, Zhang Y, Huang J, Yang Q: **Transfer Learning via Learning to Transfer.** In

*ICML*. 2018:5085-5094.

5. Gretton A, Borgwardt KM, Rasch MJ, Scholkopf B, Smola A: **A kernel two-sample test.**

*J Mach Learn Res* 2012, **13:**723-773.

6. Borgwardt KM, Gretton A, Rasch MJ, Kriegel HP, Scholkopf B, Smola AJ: **Integrating structured biological data by Kernel Maximum Mean Discrepancy.** *Bioinformatics* 2006, **22:**e49-57.
7. Gretton A, Borgwardt KM, Rasch M, Scholkopf B, Smola AJ: **A kernel method for the two-sample-problem.** In *NIPS*. 2006:513-520.
8. Ganin Y, Lempitsky V: **Unsupervised Domain Adaptation by Backpropagation.** In

*ICML*. 2015:1180-1189.

**Second round of review**

**Reviewer 1**

The authors have addressed all of my comments.

**Reviewer 2**

1. Seurat v3 was published in Cell very recently, so may be updated.

2. line 107: Compare --> Comparison of

3. line 160: Did scVI fail? In Fig S2c8, two batches are combined (north and east), though not homogeneous. Can they represent subtle biological difference? I mean 'fail' can be too strong.

4. Response for Comment 7 needs to be summarized and added in Methods section. This can be an important point for method developer.

**Authors’ response to reviewers:**

**Response to Reviewer 2**

Comment 1: Seurat v3 was published in Cell very recently, so may be updated.

*Response: We thank the reviewer for this comment. We have updated the references accordingly.*

Comment 2: line 107: Compare --> Comparison of

*Response: Thank you for this suggestion. We have revised the manuscript accordingly.*

Comment 3: line 160: Did scVI fail? In Fig S2c8, two batches are combined (north and east), though not homogeneous. Can they represent subtle biological difference? I mean 'fail' can be too strong.

*Response: Thank you for this insightful comment. In our experiment, the data simulated by Splatter should be combined homogeneously within each cell type if the batch effects are removed properly. Although scVI could align corresponding cell types, it could not remove batch effects at a more granular level to properly merge cells from different batches within each cell type. We have also revised the manuscript accordingly (Line 160-162).*

Comment 4: Response for Comment 7 needs to be summarized and added in Methods section. This can be an important point for method developer.

*Response: We thank the reviewer for this great suggestion. We have included this part in the Methods section (Line 422-442).*
